# Branch-Critical Clipping of a Ruptured Carotid–Posterior Communicating Aneurysm with Fetal PCA Configuration

**DOI:** 10.3390/diagnostics16020307

**Published:** 2026-01-18

**Authors:** Catalina-Ioana Tataru, Cosmin Pantu, Alexandru Breazu, Felix-Mircea Brehar, Matei Serban, Razvan-Adrian Covache-Busuioc, Corneliu Toader, Octavian Munteanu, Mugurel Petrinel Radoi, Adrian Vasile Dumitru

**Affiliations:** 1 Faculty of General Medicine, “Carol Davila” University of Medicine and Pharmacy, 050474 Bucharest, Romania; 2 Department of Opthamology, “Carol Davila” University of Medicine and Pharmacy, 020021 Bucharest, Romania; 3 Central Military Emergency Hospital “Dr. Carol Davila”, 010825 Bucharest, Romania; 4 Department of Anatomy, “Carol Davila” University of Medicine and Pharmacy, 050474 Bucharest, Romania; 5 Department of Neurosurgery, “Carol Davila” University of Medicine and Pharmacy, 050474 Bucharest, Romania; 6 Department of Vascular Neurosurgery, National Institute of Neurology and Neurovascular Diseases, 077160 Bucharest, Romania; 7 Puls Med Association, 051885 Bucharest, Romania; 8 Department of Pathology, Faculty of Medicine, “Carol Davila” University of Medicine and Pharmacy, 030167 Bucharest, Romania

**Keywords:** aneurysmal subarachnoid hemorrhage, posterior communicating aneurysm, fetal posterior cerebral artery, microsurgical clipping, junction reconstruction, delayed cerebral ischemia, nimodipine, transcranial Doppler, CT angiography, functional outcome

## Abstract

**Background/Objectives:** Aneurysmal subarachnoid hemorrhage (aSAH) involves a sudden onset of a perfusion-pressure injury from the initial insult combined with a secondary injury phase produced by delayed cerebral ischemia, cerebrospinal fluid circulation disturbances, and generalized instability of the patient’s physiological state. The situation may be further complicated when there has been rupture of the aneurysm at the site of the carotid–posterior communicating (PCom) artery junction that occurs in conjunction with a fetal configuration of the posterior cerebral artery (fPCA), thereby making definitive treatment dependent on preserving the critical nature of the branches of the posterior circulation since the aneurysm’s neck plane coincides with the dominant posterior circulation conduit. **Case Presentation:** A 65-year-old female patient who was obese (Grade III BMI = 42), had chronic bronchial asthma, and arterial hypertension experienced a “thunderclap” type of headache in the right retro-orbital area followed by a syncopal episode and developed acute confusion with agitation. Upon admission to the hospital, her Glasgow Coma Scale (GCS) was 13, her FOUR score was 15, her Montreal Cognitive Assessment (MoCA) score was 12/30, her Hunt–Hess grade was 3, WFNS grade 2, and Fisher grade 4 SAH with intraventricular extension. Digital subtraction angiography (DSA) and three-dimensional rotational angiography revealed a posteriorly directed right carotid communicating aneurysm that had a relatively compact neck (approximately 2.5 mm) and sac size of approximately 7.7 × 6.6 mm, with the fPCA originating at the neck plane. Microsurgical treatment was performed with junction-preserving reconstruction with skull base refinement, temporary occlusion of the internal carotid artery for a few minutes, placement of clips reconstructing the carotid–PCom interface, and micro-Doppler verification of patent vessel. Postoperatively, the blood pressure was kept within the range of 110–130 mmHg with nimodipine and closely monitored. The neurological recovery was sequential (GCS of 15 by POD 2; MoCA of 22 by POD 5). By POD 5 CT scan, the clip remained positioned in a stable fashion without evidence of infarct, hemorrhage, or hydrocephalus; at three months she was neurologically intact (mRS 0; Barthel 100; MoCA 28/30), and CTA confirmed persistent exclusion of the aneurysm and preservation of fPCA flow. **Conclusions:** In cases where the ruptured aneurysm is located at the carotid communicating junction with the PCom artery in a configuration of the posterior cerebral artery that is described as fetal, clip treatment should be viewed as a form of branch-preserving junction reconstruction of the carotid–PCom junction supported by adherence to controlled postoperative physiology and close ppostoperativesurveillance.

## 1. Introduction

Both the acute mechanical damage and blood flow compromise due to the initial rupture of the aneurysm itself, and the later secondary physiological phase of instability in cerebral blood flow, failure of auto-regulation, and altered cerebro-spinal fluid (CSF) flow patterns after successful clipping or coiling of the aneurysm contribute to the risk of a catastrophic, high-risk cerebrovascular event, such as an aneurysmal subarachnoid hemorrhage (aSAH) [1]. Although the second phase of deterioration after a SAH may occur rapidly, the risk of, and clinical evidence of, this phase depends upon the location of the rupture and how the aneurysm disrupts the cistern and its surrounding vascular structures, potentially leading to late complications, such as vasospasm. Therefore, the success of treatment of a SAH cannot be achieved by simply and rapidly excluding the aneurysm but must involve finding the balance between definitively eliminating the aneurysm and preserving the parent vessel(s), their perforators, and the architecture of the cisterns during the vasospasm-risk interval after treatment [2].

Carotid–posterior communicating (PCom) aneurysms provide a paradigmatic illustration because the surgical feasibility of these aneurysms is determined by the two independent factors of the geometric configuration of the aneurysm sac and the microsurgical anatomy of the region. Carotid–PCom aneurysms develop where the supra-clinoid internal carotid artery transitions to a densely innervated communicating segment immediately distal to the oculomotor cisternal corridor and basal forebrain-diencephalic networks and account for about one-fourth of all intracranial aneurysms. Exposure of a ruptured carotid–PCom aneurysm is made more difficult when there is a fetal-type posterior cerebral artery (fPCA); this results in the posterior cerebral territory being perfused primarily through the carotid via the PCom, and the artery that must be preserved occupies the same millimeter-scale neck plane as the aneurysm rupture site [3]; therefore, clipping is transformed from simple neck closure to reconstructive preservation of the junction. Therefore, an anatomy-based first approach that uses imaging to define the operative geometry instead of a diagnosis is necessary for the effective treatment of a ruptured carotid–PCom aneurysm: three-dimensional characterization of the true neck plane, the origin of branches and the distribution of perforators; intra-operative restoration of cisternal anatomy and minimal deformation of brain tissue due to traction; and intra-operative evaluation of laminar flow across the carotid–communicating junction [4]. These principles apply equally to the postoperative period, during which the primary risks are delayed ischemic insult and alterations in CSF flow, and standard neuro-intensive care practices, including the use of nimodipine for vasospasm prevention, remain essential [5].

In this article, we report a high-grade aSAH (Hunt–Hess 3; WFNS 2; Fisher grade 4) due to a ruptured right carotid–PCom aneurysm in which an fPCA originated at the aneurysm neck plane, creating an extremely low threshold for complete exclusion. We are not presenting this case to describe a new method but to illustrate a reproducible sequence of steps to achieve pre-operative 3D neck mapping, sequential cisternal relaxation and corridor unification, perforator-preserving junctional reconstruction, and structured neuro-intensive care to allow for long-term exclusion of the aneurysm and preservation of a major posterior circulation conduit in a critical branching condition.

## 2. Case Presentation

The patient’s history is that of a 65-year-old obese female who has been suffering from chronic bronchial asthma and hypertension. In this case, the patient experienced a sudden onset of right retro-orbital headache and subsequently lost consciousness twice prior to becoming confused and agitated. These events are consistent with a diagnosis of acute subarachnoid hemorrhage (SAH). The patient’s baseline was mRS 1. Upon admission to the hospital, her GCS was 13 (E4V4M5), and she had meningismus (significant neck stiffness, photophobia, and nausea upon slight positional changes) and severe headache (NRS 8/10). Her MoCA was 12/30 with significant impairments in both attention and executive functions (she was unable to complete serial subtraction, and also had difficulty with recall). Her FOUR score was 15 (Eye Component = 3), and she had preserved brainstem function. At the time of admission, the patient was hypoxic and hypoventilating (SpO_2_ = 94% on room air, RR = 24/min with significantly increased work of breathing, EtCO_2_ = 48 mmHg on nasal cannula); these findings indicate that she developed early hypercapnia secondary to her obesity and asthma. The patient’s blood pressure was 182/110 mmHg (MAP = 134) with significant autonomic liability (HR = 58–112 bpm). Her Hunt–Hess score was 3, and WFNS score was 2. Despite her confusion, the patient’s NIHSS was only 4, and this was largely due to her dysarthria and left lower facial weakness. Both pupils were reactive bilaterally, although she did have intermittent anisocoria (right pupil larger than left by 1–2 mm). The patient never exhibited sustained gaze deviation, and she had decreased attention/arousal, evidenced by hypometric and slow saccades to command, rather than a fixed oculomotor palsy. Given her hyperacute onset and meningeal signs, the differential diagnosis strongly suggested a primary vascular hemorrhage. However, large intraparenchymal hemorrhage was less likely based on the patient’s meningeal signs and lack of dense focal motor deficits. Postictal state was also considered but could not account for the patient’s meningeal signs. Infectious meningoencephalitis was unlikely given the patient’s afebrile status (36.7 °C) and hyperacute presentation.

**Figure 1:**
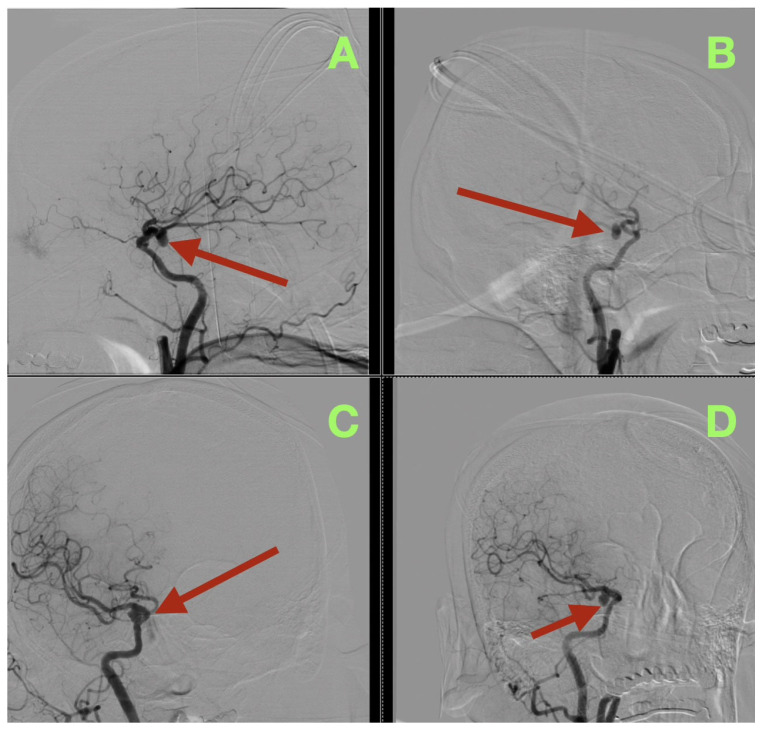
Preoperative digital subtraction angiography (DSA). (**A**) Selective right internal carotid injection in a lateral projection demonstrates a posteriorly directed saccular aneurysm arising from the communicating segment (arrow), seated at the carotid–posterior communicating region. (**B**) Oblique projection confirms posterior dome projection (arrow) and refines the profile of the aneurysm base at the junctional complex. (**C**) Complementary lateral/oblique acquisition delineates the compact neck architecture (arrow), supporting a focal saccular morphology rather than fusiform dilation. (**D**) Anteroposterior projection provides orthogonal confirmation of lesion position (arrow) at the right carotid communicating segment, validating neck definition and dome directionality across planes.

**Figure 2:**
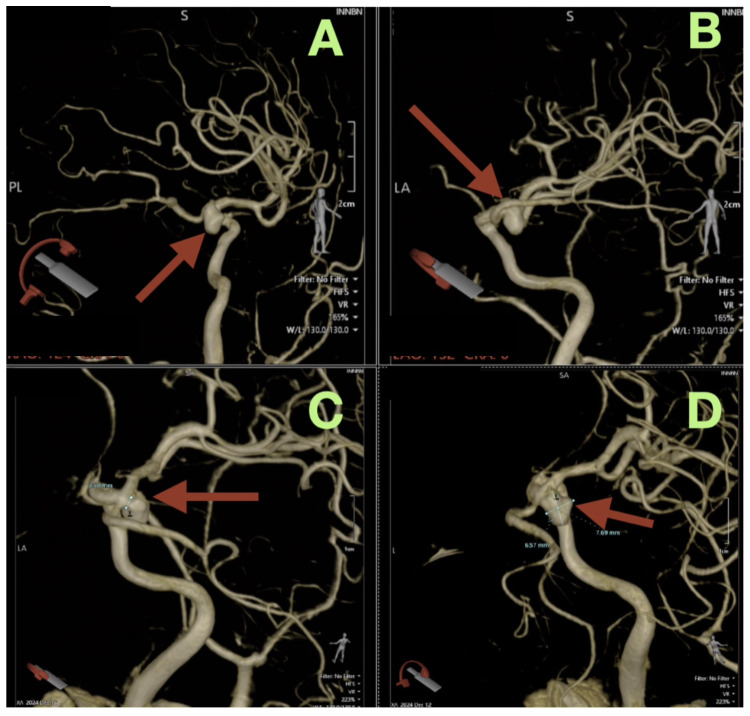
Three-dimensional rotational angiography reconstructions. (**A**) Oblique three-dimensional view demonstrates the saccular aneurysm at the right carotid communicating segment (arrow), with posterior dome projection and the aneurysm base seated at the junctional complex. (**B**) Complementary oblique view highlights the aneurysm’s relationship to the communicating region (arrow), emphasizing the shared plane between the aneurysm base and the posterior circulation outflow tract in the fetal PCA configuration. (**C**) High-magnification reconstruction provides a quantitative definition of the neck plane, demonstrating a neck width of approximately 2.5 mm (arrow). (**D**) Quantitative three-dimensional assessment demonstrates maximal aneurysm dimensions of approximately 7.7 × 6.6 mm (arrow) and reinforces the anatomic convergence of the aneurysm neck with the fetal PCA origin, the critical determinant of junction-preserving exclusion.

The first image obtained was a non-contrast cranial CT, which showed that there was an SAH (Fisher grade 4), hemorrhage into the ventricles of the brain, and generalized cerebral edema, along with evidence suggesting that the patient had acute hydrocephalus. Due to this verified hemorrhage pattern and significant risk of rebleeding, the patient underwent physiologic stabilization as part of the preoperative process; the patient was intubated and maintained a PaCO_2_ level between 35 and 38 mmHg to avoid hypercapnic related intracranial hypertension, and the patient’s arterial blood pressure was continuously monitored using nicardipine to keep the systolic blood pressure between 110 and 130 mmHg to decrease the risk of rebleeding while maintaining adequate cerebral perfusion. Selective right internal carotid artery angiography, including DSA, confirmed a saccular aneurysm located at the posterior communicating segment of the right internal carotid artery with a posteriorly oriented dome and a very narrow neck (<3 mm); the aneurysm arose from a branch critical area where a fetal type posterior cerebral artery (fPCA) branched off the aneurysm neck plane (Figure 1A–D). The presence of the aneurysm at a branch critical area made it necessary to reconstruct the junction of the aneurysm neck to the parent vessel, versus simply closing the aneurysm neck to preserve the lumen of the fPCA. Three-dimensional rotational angiography further defined the operative geometry and confirmed the aneurysm’s neck diameter to be approximately 2.5 mm and the maximum sac diameters to be approximately 7.7 × 6.6 mm (Figure 2C,D). The aneurysm was located at the carotid–PCom junction and was closely associated with the oculomotor cisternal corridor providing a potential anatomic basis for the intermittent pupillary variability observed during the acute phase of hemorrhage.

Microsurgical clipping was undertaken to address the patient’s ruptured aneurysm. Due to the patient’s reduced brain compliance and branch-critical fPCA anatomy, a standard skull-base approach was taken. Following intubation, the patient was placed into three-pin fixation and rotated 30° to the left, elevated 15°, and the zygomas were elevated to optimize skull-base access and relax the temporalis muscles. A right-sided pterional craniotomy was then performed through an interfascial dissection plane to preserve the superficial temporal artery fascial plexus and frontal branch of the facial nerve. Skull base drilling was performed to enlarge the operative corridor: the sphenoid wing was removed to the meningo-orbital band, and the meningo-orbital band was incised. An extradural anterior clinoidectomy was performed using a diamond burr to expose the carotid cave and enable superior and medial optic canal decompression, thereby merging the opticocarotid and carotid–oculomotor cisterns to limit frontal lobe retraction. Once the dura was opened, the brain was found to be pale and hypopulsatile. Initial relaxation of the brain was obtained by sharp fenestration of the medial opticocarotid cistern above the optic nerve sheath to release xanthochromic CSF. The sylvian fissure was then opened gradually from the proximal pars membranacea while preserving arachnoid planes and mobilizing clot without traction on swollen opercula. The supraclinoid ICA was then identified and followed to the communicating segment to restore the carotid–PCom anatomy. The PCom artery was found to course above the oculomotor nerve, which was partially embedded in a clot. A ~7 mm saccular aneurysm arose from the lateral ICA at the PCom junction, projected posteroinferiorly, and was adherent to arachnoid over the oculomotor nerve as it entered the cavernous sinus. The fPCA was a funnel-shaped continuation of the PCom trunk rather than a distinctly separate distal branch. Therefore, the aneurysm neck formed the lateral wall of the shared origin; as such, the aneurysm orifice and fPCA ostium occupied the same millimeter plane and required strict preservation of the fPCA lumen.

To minimize the risk of thermal damage to the oculomotor nerve and traction injuries to the surrounding arachnoid, the arachnoid covering the oculomotor nerve was preserved. Organized clot was mobilized from the dome and proximal PCom-fPCA trunk using microforceps, continuous irrigation, and minimal suction. Several thalamoperforators (0.2–0.5 mm) arising from the superior/medial PCom region were preserved by maintaining arachnoid sleeves and avoiding coagulation at their origins. Temporary proximal control was utilized due to the dome tension and shared neck-branch anatomy: a straight temporary clip was applied to the supraclinoid ICA for four minutes with MAP maintained at ~95 mmHg and propofol-induced burst suppression. With reduced dome tension, the ~2.5 mm neck was dissected free from the fPCA origin. A straight low-profile titanium clip was then placed inferolateral-to-superomedial, and aligned parallel to the ICA axis, with the blades positioned slightly beyond the neck and adjusted to rest on the medial carotid wall distal to the aneurysm and lateral to the fPCA ostium, achieving complete exclusion of the aneurysm while preserving the fPCA lumen. Micro-Doppler demonstrated a strong biphasic signal at the reconstructed junction without flow attenuation or focal stenosis. Minimal bipolar coagulation was employed to achieve hemostasis, primarily limited to the arachnoidal edges. A wide fenestration of the lamina terminalis was performed to improve postoperative CSF dynamics. The dura was closed tightly, the bone flap was secured, and the wound was closed in layers. The patient was then extubated in the operating room, followed commands, and had purposeful movement of all extremities. She was transferred to the neurointensive care unit with systolic blood pressure maintained at 110–130 mmHg. The operative endpoint was complete aneurysm exclusion with preserved patency of the ICA and fPCA origin at the shared neck plane.

Upon arrival at the neurointensive care unit, the patient underwent placement of continuous invasive monitors for blood pressure. Systolic blood pressure was maintained at 110–130 mmHg using nicardipine infusion, and hourly neurological examinations were performed, with emphasis on arousal, cranial nerves, and focal deficits. By the end of the second postoperative day, the patient’s GCS had improved to 15, and her FOUR score was 16. The patient’s intermittent anisocoria had resolved by 24 h, and she had no new oculomotor deficits. The patient’s photophobia and meningeal symptoms had significantly improved from severe to mild by the third postoperative day. The patient’s cognitive functioning had also begun to improve, as reflected by her MoCA score, which rose from 12/30 preoperatively to 22/30 by postoperative day five, with significant improvements in both attention and delayed recall. The patient had no clinical seizures, and her EEG surveillance showed no subclinical seizures. The patient was transitioned to oral nimodipine (60 mg every 4 h) on the second postoperative day and was able to tolerate the medication without compromising blood pressure goals.

**Figure 3:**
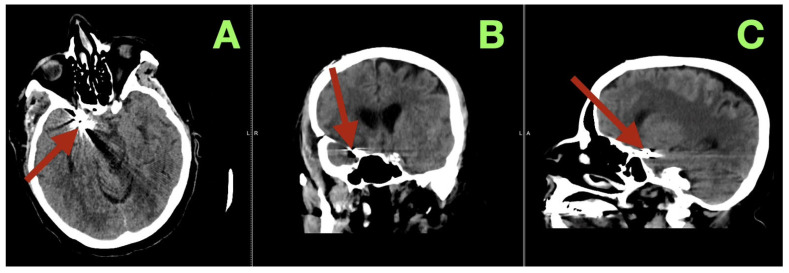
Postoperative day 5 non-contrast CT. (**A**) Axial section at the level of the skull base demonstrates the aneurysm clip at the right carotid–posterior communicating region (arrow), with expected localized metallic artifact and no evidence of recurrent subarachnoid hemorrhage, new intraparenchymal bleeding, or territorial hypodensity. The surrounding basal cisterns at this level remain discernible, without mass effect. (**B**) Coronal reconstruction confirms stable clip position within the right parasellar and suprasellar corridor (arrow), with preserved ventricular configuration and no radiographic signs of acute hydrocephalus or midline shift. (**C**) Sagittal reconstruction localizes the clip construct along the intended carotid–posterior communicating junctional plane (arrow), demonstrating anatomical coherence of the skull-base region and absence of secondary compressive changes.

During the vasospasm-risk period, serial transcranial Doppler studies of the patient’s right middle cerebral artery (MCA) velocities ranged from 85 to 105 cm/s, and her fetal PCA (fPCA) velocities ranged from 55 to 70 cm/s, indicating that there was no clinically relevant vasospasm detected sonographically. On the fifth postoperative day, non-contrast CT (Figure 3) imaging confirmed the stable position of the clip, no interval hemorrhage, no territorial hypodensities, no interval change in the configuration of the ventricles, and no interval hydrocephalus. Additionally, the basal cisterns remained open, and the basal ganglia were normal in appearance.

On the sixth postoperative day, the patient began to regain independent ambulation, and her headaches had decreased to 2/10. The patient’s wound healed uneventfully. The patient was discharged on the eighth postoperative day, with instructions to take nimodipine orally for 21 days, and to control her blood pressure strictly. At discharge, the patient’s functional status was mRS 1, and her Barthel Index was 100.

**Figure 4:**
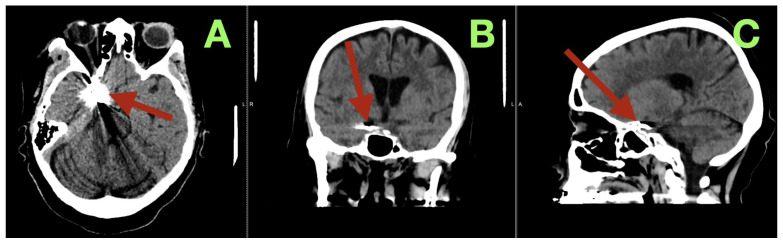
Three-month postoperative control CT angiography. (**A**) Axial source image at the level of the skull base demonstrates the aneurysm clip at the right carotid–posterior communicating junction (arrow), with stable positioning and no surrounding hypodensity, hemorrhage, or parenchymal distortion. The adjacent temporal and basal frontal regions show preserved attenuation, without signs of delayed ischemic injury. (**B**) Coronal reconstruction confirms durable anatomical restoration of the parasellar and suprasellar region (arrow), with normal ventricular configuration and no evidence of hydrocephalus, mass effect, or clip-related compromise of adjacent structures. (**C**) Sagittal reconstruction localizes the clip construct along the reconstructed carotid–posterior communicating complex (arrow), illustrating maintained skull-base anatomy, absence of clip migration, and stable postoperative remodeling of the surgical corridor.

At the three-month follow-up evaluation, the patient reported resuming all of her daily activities without experiencing recurrent severe headache, confusion, or seizure-like episodes. The patient’s neurological examination was normal (all cranial nerves intact; pupils equal/reactive; normal eye movements without ptosis/diplopia; strength 5/5; stable gait; normal coordination). The patient’s MoCA score was 28/30. The patient’s functional outcome was mRS 0, and her Barthel Index was 100. Three-month CT angiography (Figure 4A–C) demonstrated complete aneurysm exclusion, a reconstructed patent carotid–PCom junction without parent vessel stenosis, no clip migration, and robust filling of the fPCA via the preserved PCom. There was no evidence of chronic ischemia, and the CSF spaces were restored without hygroma or hydrocephalus.

Therefore, the case presented here intends to serve as a clinical instructional example, as the hemorrhagic burden and the native vascular variant converged into a singular, branch-critical problem: a ruptured right carotid–posterior communicating aneurysm that had a neck plane coinciding with the origin of a fetal-type PCA, thereby placing the rupture site and the principal conduit for posterior circulation within the same narrow reconstructive corridor. Consequently, definitive management of such lesions is influenced significantly less by aneurysm size per se but more by the geometrical aspects of the junction, the density of perforators and the need to restore a laminar, non-stenotic branch ostium while operating in the presence of unstable autoregulatory conditions associated with acute subarachnoid hemorrhage.

The value of reporting this case resides in its clear anatomical definition and in the logically replicable nature of the concepts that are inherent in the design of the operation. Definition of the neck plane using three-dimensional technology, deliberate restoration of cisternal anatomy through graded relaxation, and confirmation of parent vessel and branch patency represented essential elements of the process, as the fetal PCA variant transforms “exclusion” into junction reconstruction as opposed to mere neck closure. The postoperative course was monitored sequentially, both neurologically, using objective and quantifiable measures, and using interval imaging, and demonstrated an orderly resolution of the global SAH phenotype with no radiographic evidence of infarction, hydrocephalus progression, or junction compromise, consistent with preserved branch mechanics and stable postoperative physiology in a context in which tolerance is inherently limited.

## 3. Discussion

Although there has been advancement in our understanding of the mechanisms of injury and improved neurocritical care, the outcomes from the aSAH remain influenced by the two predominant threats: the initial hemorrhagic insult with pressure-perfusion disruption caused by rupture and the vulnerable period of time after the aSAH, when patients are at increased risk of developing delayed cerebral ischemia (DCI), hydrocephalus-related disturbances of CSF circulation, and other systemic instability [6]. Recent evidence indicates that DCI is a complex clinical–radiological syndrome that cannot be explained by angiographic vasospasm alone but rather by a series of interdependent processes including microvascular dysfunction, cortical spreading depolarizations, inflammation, disrupted autoregulatory capability, and the disruption of the blood–CSF interface barriers [7]. Table 1 outlines the continuum of injury and provides a clear indication that treatment options need to be developed to address both the immediate effects of hemorrhage and the multiple factors that contribute to secondary deterioration.

This patient demonstrates a critical rupture geometry: a carotid–PCom aneurysm with a fPCA configuration where the majority of the blood supply to the posterior cerebral territory relies upon carotid inflow, and, because of this, the potential for compensatory collaterals to maintain adequate blood flow is severely diminished [16]. Therefore, even mild compromise of the junction (e.g., clip-induced stenosis, thrombosis, vasospasm, or delayed hemodynamic compromise) could result in significant reductions in blood flow during the peak DCI-risk period. While fPCA variants occur commonly, the combination of rupture, high hemorrhage burden, and the shared neck-ostium plane of the aneurysm create a unique technical challenge, in which the primary concern is not incomplete occlusion but damage to or occult compromise of the involved branch and surrounding perforators that will not demonstrate clinical significance until the patient’s autoregulatory capabilities are compromised and their susceptibility to DCI is elevated [17,18].

Thus, the neck plane in this anatomy is not merely descriptive; it represents a three-dimensional space occupied by both the aneurysm base and the origin of the fPCA. Two-dimensional angiography can underestimate the degree of true overlap between the neck and ostium of posteriorly directed communicating aneurysms, especially those that are located in a deep basal cisternally corridor that contains perforators, cranial nerves, and clot [19]. Thus, the goal of surgery becomes junction-preserving reconstruction to restore a laminar fPCA/PCom origin and exclude the aneurysm rather than collapse the neck. Because of the inherent three-dimensional nature of this endpoint, it will require preoperative geometric mapping of the anatomic relationships and postoperative imaging verification of branch patency utilizing non-invasive methods [20].

Management post-operatively is closely related to the durability of the surgical reconstruction during the biologically unstable post-rupture interval. Current aSAH guidelines advocate for the routine use of nimodipine, as well as a structured neurointensive care approach, and various surveillance strategies, including TCD monitoring for vasospasm [21]. Interpretation of TCD data is enhanced by utilization of defined velocity thresholds, resistance ratios, and trends rather than isolated values. Surveillance should also include evaluation of the entire posterior circulation in patients whose posterior circulation is dependent on the carotid arteries via an fPCA, as clinical stability along with non-escalating TCD profiles may serve as an indirect functional marker of preserved branch patency and distal circulation tolerance during the vasospasm-risk window [22]. Furthermore, patients who develop intraventricular hemorrhage with impending CSF circulation dysfunction may experience marked cognitive-attentional impairment in the absence of destructive parenchymal injury; thus, objective bedside metrics measuring arousal, attention, and executive function may provide clinically useful indicators of recovery trajectories as meningeal inflammation and CSF dynamics improve [23].

In summary, this case combines elements of a coherent problem set: rupture at a millimeter scale of the carotid–communicating junction, fPCA anatomy that increases the consequences of junctional compromise, and high hemorrhage burden that increases secondary injury risk, managed through reconstruction-oriented clipping and branch patency assessment, as well as structured postoperative physiology and surveillance. Limitations include the single case study design and reliance on clinical examination, TCD trends, and interval imaging as surrogate markers rather than direct high-resolution flow measurements at the fPCA origin and perforator network. Additionally, the positive outcome in this case is due to the integrated microsurgical and neurocritical care pathways and thus limits attribution to specific component parts. While these anatomical principles may apply to similar configurations, they should be applied cautiously to varying geometries and levels of monitoring.

## 4. Conclusions

This particular case will illustrate how a combination of the extent of hemorrhage, post-hemorrhagic secondary injury pathophysiology, and the natural anatomical structure of the blood vessels determines the ultimate outcome from an aSAH—not solely based upon the size and morphology of the aneurysm itself. Specifically, if there exists a fetal type configuration of the PCA, then an aneurysm located in the PCoA represents a critical branching juncture of arteries, and, due to its location within the same millimeter plane of the aneurysm and the major arterial conduit of the posterior circulation, it results in a significantly decreased margin for error in terms of potential stenosis or compromise of flow during the biologically unstable time period immediately following the rupture event.

The experience described in this case illustrates a “transferable,” “anatomy first” approach: where branch–neck overlap determines the feasibility of reconstruction, the concept of definitive treatment of this critical juncture should be thought of as junction preserving reconstruction, which is facilitated through three dimensional visualization of the neck plane, deliberate restoration of cistern anatomy under conditions of reduced compliance, perforator aware microsurgical sequence, and explicit intra-operative confirmation of parent vessel and branch patency. Subsequently, the long-term success of the repair of this critical juncture is protected by a discipline of physiological corridor and trend-based surveillance, designed to limit secondary injury mechanisms extending beyond angiographic vasospasm.

We wish to draw attention to the fact that, in certain aSAH patients, in whom the vascular anatomy of the PCA is fetal-dependent, careful integration of the use of pre-operative spatial information, reconstruction-oriented clipping, and well organized neurocritical care can help protect vulnerable areas of brain tissue and facilitate successful functional recovery.

## Figures and Tables

**Table 1:** The key literature supporting physiology-informed management of aneurysmal subarachnoid hemorrhage and the branch-critical implications of fetal-type posterior cerebral artery anatomy.

Author and Year	Study Type	Population	Key Findings	Relevance to This Case
Treggiari et al. (2023) [8]	Evidence-based clinical guideline	aSAH patients (guideline scope)	Emphasizes structured neurocritical care, universal nimodipine use, and physiology-centered prevention of secondary injury during the DCI window; reinforces that outcome hinges on both early rupture injury and delayed complications.	Supports the postoperative strategy used here (tight hemodynamic corridor, nimodipine, close neurochecks), particularly important when branch-critical reconstruction must remain patent through the biologically unstable post-rupture phase.
Abdulazim et al. (2022) [9]	Multidisciplinary consensus statement	aSAH consensus definitions	Standardized the clinical–radiographic definition of delayed cerebral ischemia, distinguishing DCI from angiographic vasospasm and enabling reproducible reporting across studies.	Provides the reporting framework for interpreting the vasospasm-risk period and justifies presenting Doppler/imaging trends as supportive rather than definitive endpoints.
Nimmo et al. (2025) [10]	Narrative review (pathophysiology synthesis)	aSAH literature	Details DCI as a multifactorial syndrome (microvascular dysfunction, impaired autoregulation, inflammation, cortical spreading depolarizations, BBB/CSF disturbances), not reducible to large-artery spasm alone.	Aligns with this case’s “global SAH phenotype” and supports why meticulous junction preservation must be coupled with disciplined ICU physiology to protect distal territories.
Al-Mifti et al. (2021) [11]	Neurocritical Care Society guideline	aSAH critical-care management	Provides ICU-oriented recommendations (neuromonitoring, prevention/management of DCI, systemic complication control) and reinforces protocolized care during the high-risk window.	Supports the case’s structured ICU pathway (hourly exams, BP corridor, nimodipine, Doppler surveillance) as a coherent extension of operative reconstruction.
Davidoiu et al. (2023) [12]	Imaging anatomy study	Patients undergoing vascular imaging (Circle of Willis variants)	Characterizes fetal-type PCA variants and their anatomical implications for posterior territory inflow dependence on the carotid system.	Grounds the central anatomic premise of this report: in fPCA anatomy, even subtle junctional compromise at the carotid–PCom complex may carry disproportionate territorial consequence, making “exclusion” functionally a reconstructive task.
Han et al. (2025) [13]	Observational association study	Patients evaluated for PCom aneurysms/vascular variants	Evaluates whether ipsilateral fetal-type PCA is associated with posterior communicating artery aneurysms, supporting a hemodynamic–anatomic relationship between variant anatomy and aneurysm biology.	Reinforces why documenting fPCA is not incidental: it may relate both to aneurysm formation patterns and to the narrow patency tolerance that shaped clip strategy in this case.
Yamada et al. (2019) [14]	Technical microsurgical report (“How I do it”)	Skull-base aneurysm microsurgery	Describes extradural anterior clinoidectomy/optic canal decompression as exposure-refining maneuvers that reduce depth and improve clip vectors in paraclinoid–supraclinoid carotid aneurysm surgery.	Supports the skull-base logic used here (clinoid/optic canal work to unify cisternal corridors and avoid fixed retraction), particularly relevant in low-compliance brains after high-grade SAH.
Kuo et al. (2021) [15]	Literature synthesis (via handbook chapter)	aSAH/hydrocephalus context	Discusses lamina terminalis fenestration as a maneuver used by some groups to influence CSF dynamics and potentially reduce shunt-dependent hydrocephalus after SAH, while acknowledging heterogeneous evidence.	Provides literature context for including wide lamina terminalis fenestration in a Fisher-grade hemorrhage phenotype with hydrocephalus tendency, framed as a physiology-supporting step rather than a guarantee.

## Data Availability

The data presented in this study are available upon request from the corresponding author.
